# Structure and immunogenicity of pre-fusion-stabilized human metapneumovirus F glycoprotein

**DOI:** 10.1038/s41467-017-01708-9

**Published:** 2017-11-16

**Authors:** Michael B. Battles, Vicente Más, Eduardo Olmedillas, Olga Cano, Mónica Vázquez, Laura Rodríguez, José A. Melero, Jason S. McLellan

**Affiliations:** 1 Department of Biochemistry and Cell Biology, Geisel School of Medicine at Dartmouth, Hanover, NH 03755 USA; 2 Unidad de Biología Viral, Centro Nacional de Microbiología and CIBER de Enfermedades Respiratorias, Instituto de Salud Carlos III, 28220 Madrid, Spain; 3 Present Address: University of Rochester Medical Center, Rochester, NY 14642 USA

## Abstract

Human metapneumovirus (hMPV) is a frequent cause of bronchiolitis in young children. Its F glycoprotein mediates virus–cell membrane fusion and is the primary target of neutralizing antibodies. The inability to produce recombinant hMPV F glycoprotein in the metastable pre-fusion conformation has hindered structural and immunological studies. Here, we engineer a pre-fusion-stabilized hMPV F ectodomain and determine its crystal structure to 2.6 Å resolution. This structure reveals molecular determinants of strain-dependent acid-induced fusion, as well as insights into refolding from pre- to post-fusion conformations. A dense glycan shield at the apex of pre-fusion hMPV F suggests that antibodies against this site may not be elicited by host immune responses, which is confirmed by depletion studies of human immunoglobulins and by mouse immunizations. This is a major difference with pre-fusion F from human respiratory syncytial virus (hRSV), and collectively our results should facilitate development of effective hMPV vaccine candidates.

## Abstract (web summary)

An efficient vaccine for human metapneumovirus (hMPV) will likely rely on neutralizing antibodies against the fusion protein (F). Here, the authors determine the crystal structure of pre-fusion-stabilized hMPV F and identify a dense glycan shield that affects generation of neutralizing antibodies.

## Introduction

Human metapneumovirus (hMPV) was first isolated in 2001 by van den Hoogen et al.^1^, from children with respiratory infections from which the etiological agent had not been identified. Compelling evidence accumulated since then indicates that hMPV is a common cause of acute lower respiratory tract infections, only somewhat less frequent than human respiratory syncytial virus (hRSV), in children under 5 years of age^2–5^. Both hMPV and hRSV are also a frequent cause of morbidity and mortality in the elderly^6, 7^ and immunocompromised adults^8^. Indeed, hMPV and hRSV share not only clinical symptoms but also biological traits that led to their recent classification in the metapneumovirus and orthopneumovirus genera, respectively, of the newly created Pneumoviridae family, detached from the original Paramyxoviridae family^9^.

The hMPV genome is a single-stranded negative-sense RNA molecule that encodes nine different gene products, including three glycoproteins: G, F, and SH^10^. G and F are the main protein constituents of the virus envelope. It was originally thought that G mediated the initial interaction of hMPV virions with cell-surface proteoglycans^11^, whereas F acted at a subsequent step to promote fusion of the viral and cell membranes and hence entry of the viral ribonucleoprotein into the cell. However, the precise pathway of hMPV entry into cells is still a matter of debate. On the one hand, the G glycoprotein, as in the case of hRSV, is dispensable in recombinant hMPV for virus infectivity in vitro^12^ and for replication in the upper respiratory tract of non-human primates^13^. In addition, the F glycoprotein can interact with proteoglycans to overcome loss of the G protein in ∆G viruses^14^. On the other hand, Cseke et al.^15^ provided initial evidence that hMPV F contains a highly conserved RGD motif characteristic of proteins that bind integrins and that integrins could be functional receptors of hMPV F. Further studies, however, suggested that the interaction of hMPV F with integrins occurs after an initial binding to proteoglycans and that integrins promote hMPV infectivity by a still ill-defined mechanism^14^. Certain RGD mutants are nevertheless functional, adding extra complexity to the functional relevance of integrins in the hMPV infectious cycle^14, 16^.

The F glycoprotein is synthesized as an inactive precursor, F_0_, that requires proteolytic processing to become fusion competent. Whereas the hRSV F_0_ precursor is cleaved twice inside the cell at two polybasic sites recognized by furin-like proteases^17, 18^, the hMPV F_0_ precursor is cleaved only once by trypsin-like proteases outside the cell^19^, as is the case for the precursor of most paramyxovirus F proteins. Cleavage generates two subunits, F_2_ and F_1_, that remain covalently linked by disulfide bonds. The mature hMPV F is a trimer of disulfide-linked heterodimers that is incorporated into virions in a metastable pre-fusion conformation. During membrane fusion, the F glycoprotein refolds through a series of unstable intermediates into a highly stable post-fusion conformation^20^. hMPV F membrane fusion is enhanced in a minority of viral strains by exposure to acidic conditions^21, 22^. Therefore, although low pH is probably not a general mechanism for activation of hMPV F, studies of low pH dependency have identified regions of hMPV F that might be critical for the structural rearrangements that occur during membrane fusion^23–25^.

Recent determination of hRSV F crystal structures, folded in either the pre-fusion^26–28^ or post-fusion conformation^29, 30^, revealed the drastic metamorphosis that F undergoes during membrane fusion. Among other changes, the pre-fusion-to-post-fusion transition includes refolding of heptad repeat A (HRA) sequences of the F_1_ subunit into one long α-helix, and insertion of the fusion peptide—located at the N-terminus of HRA—into the target-cell membrane. Refolding of this fusion intermediate promotes assembly of HRA and HRB sequences into a stable six-helix bundle that drives membrane fusion and is characteristic of post-fusion F. In addition, the structural studies with hRSV F identified antigenic sites unique to the pre-fusion conformation^28, 31^ that are recognized by potent neutralizing antibodies that account for most of the neutralizing activity found in human serum^32, 33^. Antibody-mediated hRSV neutralization is thought to involve blockade of the structural changes that occur in the F protein during membrane fusion, in agreement with the high neutralizing potency of antibodies that bind unique epitopes of pre-fusion hRSV F. However, there are also neutralizing antibodies that bind epitopes shared by pre-fusion and post-fusion hRSV F, explaining the capacity of purified post-fusion hRSV F to induce neutralizing antibodies and afford protection against hRSV challenge in mice^30^.

Recently, the structure of a soluble form of post-fusion hMPV F was determined, revealing extensive similarity with post-fusion hRSV F despite having only ~38% sequence identity^34^. The purified post-fusion hMPV F protein was able to elicit high titers of neutralizing antibodies in mice, suggesting that it may be a promising vaccine candidate^34^. Although a few monoclonal antibodies capable of neutralizing both hRSV and hMPV have been reported^34–36^, no significant cross-neutralization was detected in polyclonal antibody responses elicited by soluble post-fusion forms of either hRSV F or hMPV F^34^. While the responses to a pre-fusion form of hMPV F may be different than to a post-fusion form, it is likely that a pan-pneumovirus vaccine will require two antigens, or a single chimeric antigen displaying antigenic sites from each virus.

To advance our understanding of hMPV F structure, function, and immunogenicity, we stabilized a soluble form of the hMPV F ectodomain in its pre-fusion conformation, following strategies used previously for hRSV F. After crystallization, the structure was determined by X-ray diffraction analysis to a resolution of 2.6 Å. The structure is generally similar to that of pre-fusion hRSV F, but has unique features that bestow substantial functional and immunological differences between the F proteins of hMPV and hRSV, with important implications for virus entry and vaccine development.

## Results

### Stabilization of hMPV F in the pre-fusion conformation

Based on the available structures of hRSV F, three different strategies were attempted to stabilize the analogous hMPV F ectodomain (residues 1–489) in its pre-fusion conformation. All such constructs had a foldon trimerization domain and a 6xHis-tag appended at the C-terminus. The first strategy was based on introducing a proline residue into the loop connecting α4 and α5 to prevent the coil-to-helix refolding of this region that occurs during transit from the pre-fusion to post-fusion conformation^26^. Cleavage-site modifications were also tested in the context of this strategy (Supplementary Table 1). Three residues, Ala185, Ile184, and Asp186 in hMPV F were individually substituted with proline (variants 115-BV, 116-BV, and 117-BV, respectively), since there was ambiguity about the exact sequence—structure correspondence with hRSV F (Supplementary Table 1). The best-expressed variant from transfected CV-1 cells was 115-BV, as measured by reactivity with monoclonal antibody (mAb) MF14, an antibody that recognizes an epitope equivalent to hRSV F antigenic site II, which is shared by pre-fusion and post-fusion structures. In addition, 115-BV showed poor binding to the post-fusion-specific mAb MF1 but good binding to the pre-fusion-specific mAb MPE8—characteristics indicative of a pre-fusion conformation. Hence, further development of 116-BV and 117-BV was discontinued. As proper proteolytic processing is critical for fusion activation, other variants with the A185P substitution and various cleavage-site modifications were tested. Some were either poorly expressed (134-BV), showed low reactivity with MPE8 (134-BV), high reactivity with MF1 (137-BV), or were not fully cleaved (135-BV and 136-BV), and thus their development was discontinued. Variant 130-BV showed antigenic characteristics of pre-fusion F and contained the same A185P substitution as 115-BV, but the cleavage site of this variant was designed to remain unprocessed by furin-like proteases.

The second strategy was to introduce amino acid changes that would fill cavities observed in the structure of pre-fusion hRSV F bound to mAb D25^28^. The substitution A161F (119-BV) prevented expression of hMPV F, whereas the A161L substitution (120-BV) resulted in F protein that reacted poorly with MPE8, suggesting that it was not in the pre-fusion conformation (Supplementary Table 1). In contrast, variant 118-BV, with the substitution F456W, was expressed to moderate levels and reacted poorly with MF1 but relatively well with MPE8, indicative of pre-fusion antigenic characteristics. The third strategy for pre-fusion stabilization of the hMPV F ectodomain was to introduce intra-chain disulfide bonds, as was done with hRSV F^27^. However, the three disulfide variants (121-BV, 122-BV, and 123-BV) were not expressed at levels sufficient for western blot or ELISA (enzyme-linked immunosorbent assay) detection.

**Fig. 1:**
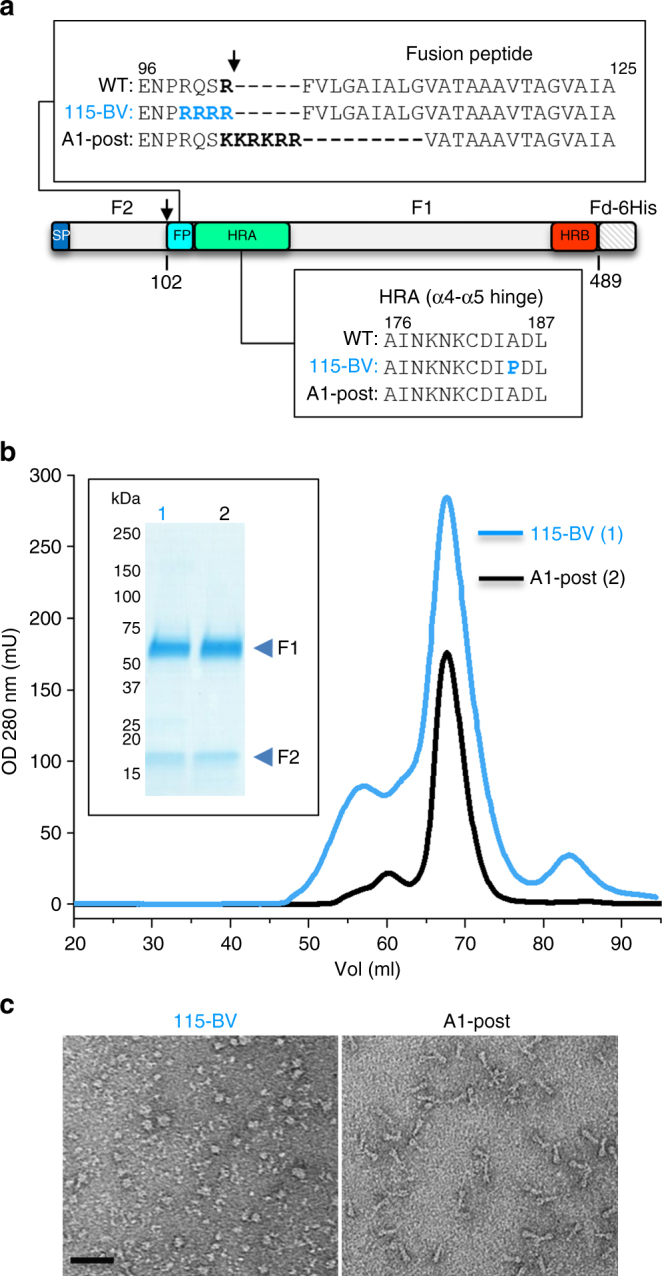
Purification and characterization of the pre-fusion hMPV F, 115-BV. **a** Diagram of the hMPV F ectodomain primary structure (NL/1/00 strain, A1 sublineage), denoting the signal peptide (SP), the fusion peptide (FP), and the heptad repeats (HRA and HRB). The proteolytic cleavage site that generates the F_2_ and F_1_ subunits is indicated by an arrow. Sequence changes in the cleavage site and HRA sequences are shown above and below the protein diagram, respectively, for 115-BV (pre-fusion) and A1-post (post-fusion) proteins. **b** Gel-filtration traces of 115-BV and A1-post, as indicated. Inset shows a Coomassie blue-stained SDS-PAGE, run under reducing conditions, of the major peak of each chromatogram. **c** Electron microscopy of the indicated negatively stained proteins. Scale bar: 50 nm

Therefore, variants 115-BV, 130-BV, and 118-BV, all of which had antigenic characteristics of pre-fusion hMPV F, were chosen for further development. Vaccinia viruses expressing each of the indicated proteins were generated to enable large-scale protein production and purification from supernatants of CV-1 cells. Variant 115-BV, as well as the uncleaved variant 130-BV, were purified to higher levels than post-fusion F (Fig. 1a, b) and retained antibody-reactivity characteristics of pre-fusion hMPV F (Supplementary Table 2). In contrast, the yield of purified 118-BV was low and its reactivity with MPE8 decreased after purification, indicative of instability.

Based upon these results, 115-BV was chosen as the best candidate for a cleaved soluble form of pre-fusion hMPV F and was subjected to additional characterization. Electron micrographs of negatively stained 115-BV displayed round molecules that resembled pre-fusion hRSV F^28^ and contrasted with the cone-shaped molecules of post-fusion hMPV F (Fig. 1c). To quantitatively assess its antigenic properties, the reactivity of 115-BV with antigen-binding fragments (Fabs) of conformation-specific antibodies was assessed by surface plasmon resonance (Table 1 and Supplementary Fig. 1). The affinity (*K*_D_) of 115-BV for MF14 and MF16 was about threefold weaker than the affinity of post-fusion hMPV F protein (A1-post) for those same antibodies. However, the most substantial differences were seen with Fabs derived from MF1, MPE8, and ADI-15614. The post-fusion-specific MF1 Fab bound efficiently to A1-post but showed no binding with 115-BV. In contrast, mAbs MPE8 and ADI-15614 Fabs, which bind preferentially to the pre-fusion conformation^31, 35^, showed considerable binding with 115-BV but no binding with A1-post. Collectively, these results indicate that 115-BV retains the pre-fusion conformation after large-scale expression and purification. These data also suggest that the proline substitution strategy is more appropriate than cavity-filling and disulfide bond strategies for stabilization of class I fusion proteins when high-resolution structural information is unavailable.

**Table 1:** Surface plasmon resonance experiments

Fab	Protein	*K*_D_ (nM)	*k*_a_ (M^−1^ s^−1^)	*k*_d_ (s^−1^)
MF14 (site II)	115-BV	9.65	1.80 × 10^5^	1.73 × 10^−3^
	A1-post	3.85	4.48 × 10^5^	1.73 × 10^−3^
MF16 (site IV)	115-BV	54.5	1.33 × 10^5^	7.25 × 10^−3^
	A1-post	18.1	1.88 × 10^5^	3.41 × 10^−3^
MF1 (anti-6HB)	115-BV	ND	ND	ND
	A1-post	7.94	5.51 × 10^5^	4.37 × 10^−3^
MPE8 (site III)	115-BV	64.8	2.28 × 10^5^	1.48 × 10^−4^
	A1-post	ND	ND	ND
ADI-15614 (site III)	115-BV	52.3	2.00 × 10^5^	1.05 × 10^−4^
	A1-post	ND	ND	ND

ND, Not determined; Biacore experiments were performed but no binding was detected

### Crystal structure of pre-fusion hMPV F

Crystals of 115-BV were obtained in space group *I*2_1_3 and diffracted X-rays to 2.6 Å resolution. A molecular replacement solution was obtained using a hybrid search model consisting of a portion of the post-fusion hMPV F structure (PDB ID: 5L1X)^34^ and a high-resolution structure of pre-fusion hRSV F (PDB ID: 5C69)^26^. The asymmetric unit contained a single protomer of pre-fusion hMPV F, with the trimer axis aligned along the threefold crystallographic axis. Iterative rounds of model-building and refinement produced a final structure with an *R*_work_/*R*_free_ of 17.0/20.7% (Supplementary Table 3).

**Fig. 2:**
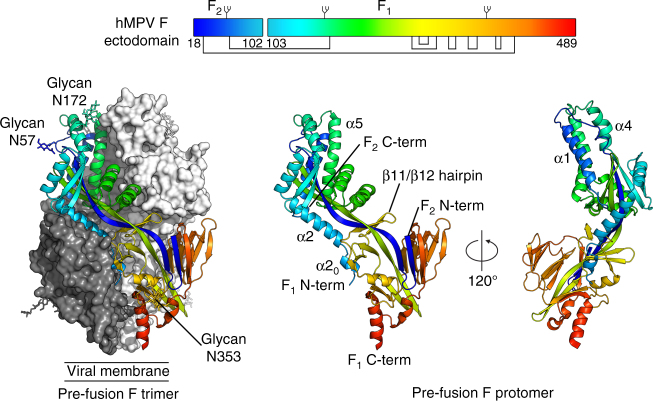
Structure of pre-fusion hMPV F. (Left) hMPV F crystal structure shown as the biological trimer. One protomer is shown as ribbons and colored as a rainbow from blue to red, N-terminus of F_2_ to C-terminus of F_1_, respectively. Molecular surfaces are shown for the other two protomers, colored white and dark gray. *N*-linked glycans observed in the structure are shown as sticks. (Middle) A single hMPV F protomer in ribbon representation. (Right) 120° rotation of the protomer about the vertical axis. Inset: schematic of the mature hMPV F glycoprotein ectodomain. The rainbow coloring matches the structures. Glycans are shown as branches and disulfide bonds are shown as black lines, above and below the primary structure, respectively

The structure contains F_2_ residues 19–90 and F_1_ residues 103–472 (Fig. 2). The signal sequence (residues 1–18) was removed intracellularly during protein expression, and electron density for residues 473–489 and the C-terminal foldon trimerization domain was not observed, likely due to flexibility of this region, consistent with what has been observed in pre-fusion structures of hRSV F^26, 28^. Similarly, the C-terminal residues of F_2_ (91–102) were not observed and are expected to project out away from the protein into the solvent. Electron density was observed, however, for glycans attached to the three *N*-linked glycosylation sites at asparagine residues 57, 172, and 353 (Supplementary Fig. 2), as explained in further detail below. The shape of the trimeric pre-fusion hMPV F protein is spheroidal, consistent with the negatively stained micrographs in Fig. 1c. In comparison to the post-fusion hMPV F structure^34^, residues at the N- and C-termini of F_1_ are in dramatically different positions (Supplementary Fig. 3), as expected based on the mechanism of F-mediated membrane fusion and the structures of other F glycoproteins^37, 38^. Overall, the structure of pre-fusion hMPV F is highly similar to pre-fusion hRSV F (RMSD of 1.4 Å for 428 Cα residues), despite relatively low protein sequence identity (38%) of the mature ectodomains (Supplementary Fig. 4a). Most individual domains were also similar to a partial monomeric structure of hMPV F bound to a neutralizing antibody^39^ (Supplementary Fig. 4b).

### Structural insights into fusion initiation and refolding

**Fig. 3:**
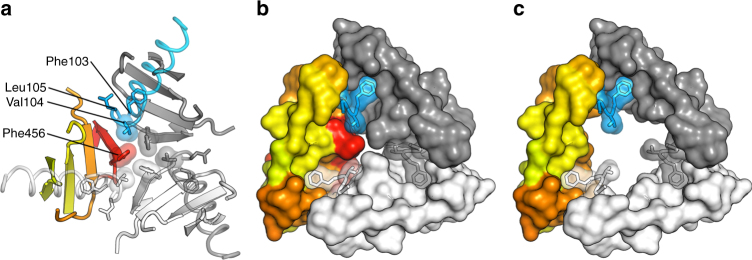
Fusion peptides reside inside the trimer in a hydrophobic cavity. **a** Center of the pre-fusion F protein viewed down the threefold axis toward the viral membrane. Coloring is the same as in Fig. 2. The fusion peptides are represented as semi-transparent helical coils. The side chains of the three N-terminal residues of the fusion peptide, along with Phe456 in the HRB, are shown as sticks. **b** Molecular surfaces are shown for the regions interacting with the N-termini of the fusion peptides. **c** Same as **b**, except residues in HRB have been omitted

The fusion peptide of pre-fusion hMPV F is buried inside the central cavity of the trimer (Fig. 3a), similar to what has been observed for pre-fusion hRSV F^28^, and in contrast to the external location observed for paramyxovirus pre-fusion F proteins from PIV5, Hendra, and Nipah^40–42^. Interestingly, the N-terminus of the hMPV fusion peptide (Phe103) nestles inside a hydrophobic cavity of a neighboring protomer, whereas Leu105 resides inside a hydrophobic cavity formed primarily within the same protomer (Fig. 3b). Both residues, and thus the fusion peptide as a whole, appear to be trapped in this conformation due to an interaction of Val104 with the central Phe456 residues of HRB. We thus speculate that for pre-fusion hMPV F to transition to the post-fusion state, this HRB region must be destabilized to vacate the central cavity and release the fusion peptide (Fig. 3c).

**Fig. 4:**
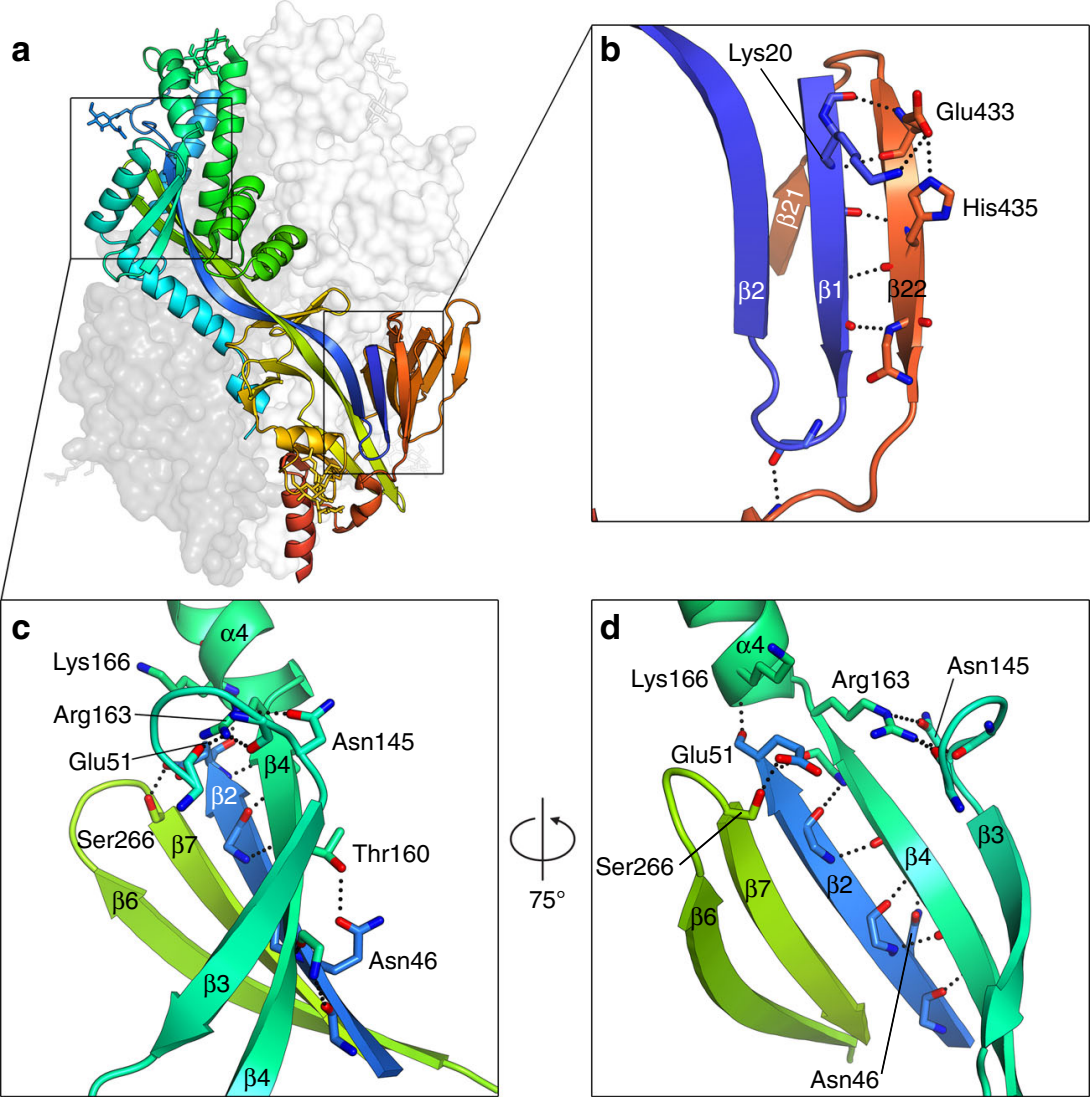
Parallel β-strands dissociate during fusion. **a** Side view of pre-fusion hMPV F depicted as in Fig. 2 with white and dark gray protomers shown as transparent molecular surfaces. **b** Magnified view of the putative pH sensor. **c** Magnified view of the 5-strand β-sheet involving part of HRA. **d** 75° rotation of **c** about the vertical axis. For all panels, select side chains are shown as sticks, as are main-chain atoms contributing to interactions between parallel β-strands. Oxygen atoms are colored red and nitrogen atoms are colored blue. Hydrogen bonds and salt bridges are shown as black dotted lines

Low pH can enhance the fusion activity of hMPV F in certain strains^21, 22^ (including NL/1/00 used in this study) and it has been suggested that protonation of a conserved histidine residue (His435) just N-terminal to HRB is important for this effect^23–25^. Our structure reveals that His435 is located in a parallel β-strand (β22) adjacent to Lys20 on β1, forming one of only two parallel β-strand interactions in the pre-fusion hMPV F structure (Fig. 4a, b). At physiological pH (7.4), Lys20 would be expected to form a salt bridge with Glu433, stabilizing the parallel β1–β22 interaction. At low pH, however, protonation of His435 would be predicted to destabilize this interaction through a histidine–cation electrostatic repulsion mechanism^43^. We postulate that this may lead to dissociation of β22, rearrangement of HRB, and removal of Phe456 from the central cavity. In support of this model, mutation of His435 to positively charged residues (Lys or Arg) was shown to retain the acidic-fusion phenotype, whereas mutation to negatively charged residues at this position resulted in substantially reduced fusion at both low and neutral pH, presumably due to increased stability of the pre-fusion conformation^23, 24^. However, protonation of this sensor, in addition to other changes resulting from acidification, may lead to global destabilization and triggering of pre-fusion hMPV F without transition through the sequence of events suggested above.

The only other parallel β-strand interaction in the structure involves β2 and β4, and like β22, β4 must dissociate and undergo a large structural rearrangement during the fusion process. Indeed, the transition for β4 is even more dramatic, because it, along with β3, refolds into part of an elongated α-helix in the post-fusion structure (Supplementary Fig. 3). The 5-strand β-sheet containing β3 and β4 is near a dense electrostatic region containing positively charged Arg163 and Lys166 in F_1_ and negatively charged Glu51 in F_2_. Arg163 occupies a central position in this region, and makes extensive polar contacts with the α3-β3 loop (Fig. 4c, d). In this way, the position of Arg163 helps coordinate the region of pre-fusion HRA secondary structure that is dramatically altered during the pre-fusion-to-post-fusion transition. In addition, Glu51 is adjacent to Arg163, and has been shown in previous studies to be a critical residue for proper folding, cleavage activation, and fusogenicity of hMPV F^23^. Collectively, these observations reveal that refolding of hMPV F to the post-fusion conformation involves two electrostatic regions that contain parallel β-strand interactions that must dissociate.

### The RGD integrin-binding motif

In the pre-fusion hMPV F structure, the conserved arginine–glycine–aspartic acid (RGD) motif is located in the β11–β12 hairpin at an inter-protomeric interface (Supplementary Fig. 5a, b). However, the RGD motif is largely buried by the neighboring protomer (Supplementary Fig. 5c), in contrast to previous homology modeling studies using pre-fusion hRSV F as a template^16^. In particular, Arg329 is almost completely buried by the neighboring protomer, and the side chain is oriented toward the central cavity, lying underneath a protective shelf composed of the C-terminus of F_2_ (Supplementary Fig. 5d). The orientation and buried nature of the RGD motif suggests that it would not be accessible to RGD-binding integrins in this conformation. Similarly, in the post-fusion conformation, the RGD motif remains partially buried despite a reorientation of the Arg329 side chain (Supplementary Fig. 6). Thus, for RGD-binding integrins to bind hMPV F, a conformational change involving separation of the protomers is likely required.

### Glycan masking of a pre-fusion-specific antigenic site

**Fig. 5:**
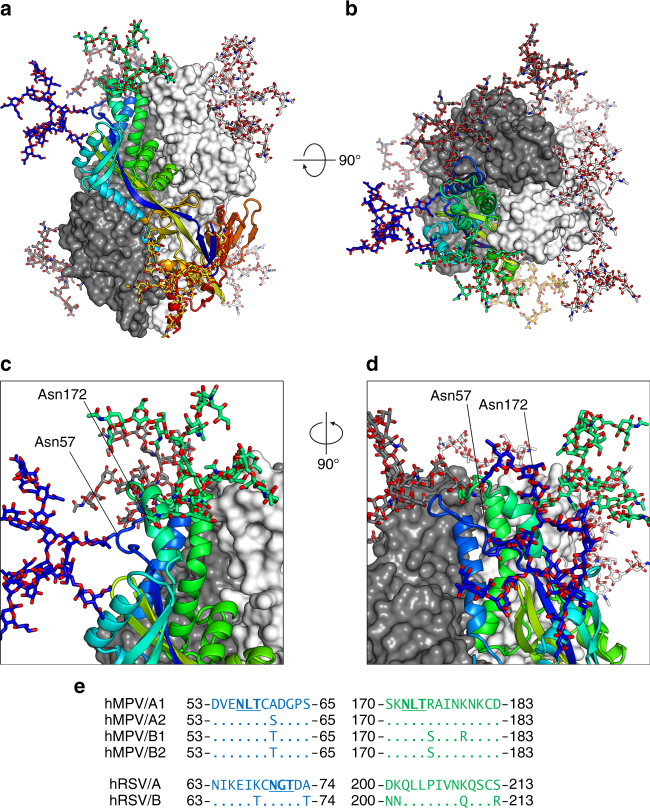
Conserved *N*-linked glycosylation shields the apex of pre-fusion hMPV F. **a** Side view and (**b**) top view of pre-fusion hMPV F with complex glycans (modeled in silico via GlyProt^70^) shown as sticks. **c** Magnified view of the apex, and (**d**) 90° rotation about the vertical axis. **e** Alignment of antigenic site Ø for prototypic hMPV and hRSV strains, colored to match the cartoon representations. Only amino acid changes with respect to the hMPV A1 and hRSV A lineage are shown. Dots represent amino acid identity. *N*-linked glycosylation sequences are underlined and in bold

Electron density corresponding to *N*-linked glycans was observed at each of the three predicted glycosylation sites: a single *N-*acetylglucosamine (GlcNAc) was observed at Asn57, both core GlcNAcs were observed at Asn172, and a Man_2_GlcNAc_2_ glycan was observed at Asn353 (Fig. 2 and Supplementary Fig. 2). Because the protein was produced in CV-1 cells, and no endoglycosidase-treatment was performed prior to crystallization, it is likely that complex glycans are present at each site, although electron density for the remaining glycan moieties is not observed due to disorder. Analysis of a panel of 91 hMPV F protein sequences from the online Virus Pathogen Resource database revealed that all three glycosylation sites are completely conserved. One well-established role for *N*-linked glycosylation of viral envelope glycoproteins is the modulation of host antibody responses through shielding of neutralization-sensitive epitopes^44–46^. The pre-fusion hMPV F structure reveals that Asn57 and Asn172 are located in an antigenic site near the trimer apex (site Ø, “zero”), which in hRSV F is the target of potently neutralizing pre-fusion-specific antibodies^28, 31, 47, 48^. hRSV F, however, contains only a single *N*-linked glycan site in this region, and it is located closer to the trimer apex (Supplementary Fig. 7). Based on modeling of complex glycans at each site on pre-fusion hMPV F, we predict that site Ø would be masked from antibody recognition (Fig. 5), and that site Ø-directed pre-fusion-specific antibodies would be rarely elicited by natural infection with hMPV or vaccination with natively glycosylated hMPV F antigens.

### hRSV F and hMPV F elicit different antibody specificities

**Fig. 6:**
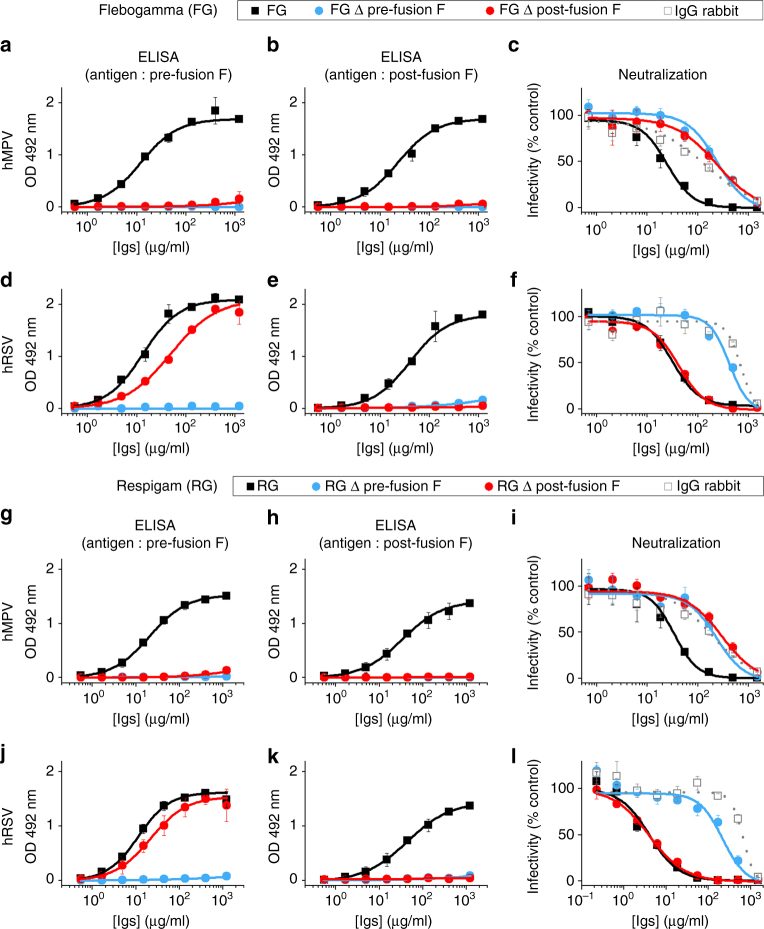
Depletion of Flebogamma and RespiGam with pre-fusion- and post-fusion-stabilized hMPV and hRSV F glycoproteins. **a**–**f** Flebogamma (FG, upper half) and (**g**–**l**) RespiGam (RG, lower half) Igs were pre-incubated for depletion of antibodies that bind to either pre-fusion (∆ pre-fusion F) or post-fusion (∆ post-fusion F) forms of hMPV F or hRSV F (indicated at left of panels). The original and depleted Igs were tested for ELISA binding to either pre-fusion or post-fusion forms of hMPV F or hRSV F as indicated above left and middle panels. The original and depleted Igs were also tested for neutralization of hMPV (upper right panels) and hRSV (lower right panels), as shown in upper and lower halves, using a naive rabbit IgG preparation as negative control. Note the different *x* axis used for hRSV neutralization performed with RG. Results are presented as means ± SD of three independent experiments

To test the hypothesis that hMPV infection may produce lower titers of pre-fusion-specific antibodies as compared to hRSV, antibodies of this type were evaluated in two human immunoglobulin (Ig) preparations. Flebogamma (FG) is a normal Ig preparation obtained from unselected donors whereas RespiGam (RG) is an Ig preparation obtained from donors screened for high titers of hRSV-neutralizing antibodies. RG was used prophylactically to prevent severe hRSV infections in high-risk children^49^, but both preparations contain hMPV-specific neutralizing antibodies. Depletion of FG or RG with pre-fusion hMPV F (115-BV) removed all antibodies capable of binding not only to pre-fusion hMPV F (Fig. 6a, g) but also to post-fusion hMPV F (Fig. 6b, h). Similarly, depletion of FG and RG with post-fusion hMPV F eliminated antibody binding to the post-fusion (Fig. 6b, h) and pre-fusion conformations of hMPV F (Fig. 6a, g). In contrast, depletion of FG and RG with post-fusion hRSV F removed the antibodies binding to post-fusion F (Fig. 6e, k), but did not eliminate the majority of antibodies binding to pre-fusion hRSV F (Fig. 6d, j). Those presumable pre-fusion-specific antibodies were only removed after depletion with the pre-fusion form of hRSV F (Fig. 6d, j). Hence, the hMPV F-reactive antibodies in FG and RG do not discriminate between pre-fusion and post-fusion hMPV F, whereas the majority of hRSV F-reactive antibodies in those Ig preparations bind preferentially to pre-fusion hRSV F.

To assess the impact of the antibody depletions on virus neutralization, FG and RG were tested in microneutralization assays either before or after depletion. Depletion of FG or RG with either the pre-fusion or post-fusion form of hMPV F removed most of the neutralizing activity against this virus (Fig. 6c, i), shifting the neutralization curves toward that of naive rabbit serum, used as a negative control. In contrast, depletion of FG or RG with post-fusion hRSV F had essentially no effect on neutralization of hRSV. Only depletion with pre-fusion hRSV F removed the neutralizing activity against this virus (Fig. 6f, l). These results indicate that the fraction of antibodies binding specifically to pre-fusion hRSV F represent the majority of the hRSV-neutralizing activity present in FG and RG. In contrast, hMPV-neutralizing antibodies of the two Ig preparations did not discriminate between pre-fusion and post-fusion F.

**Fig. 7:**
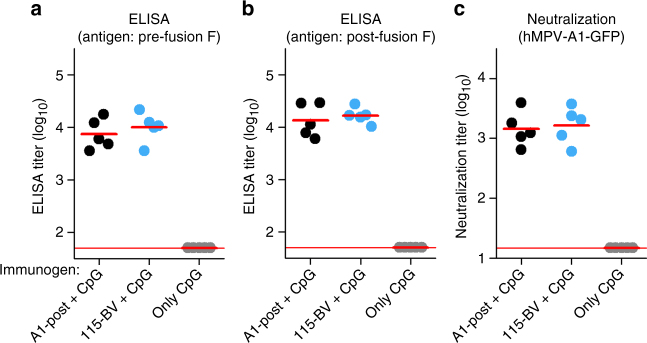
Immunogenicity of pre-fusion and post-fusion hMPV F in mice. BALB/c female mice (6-week-old) were inoculated twice (4 weeks apart) with 10 µg each of either post-fusion (A1-post, black dots) or pre-fusion (115-BV, blue dots) hMPV F mixed with CpG. Negative-control mice were inoculated with CpG only (gray dots). Two weeks after the last inoculation, serum was drawn and tested for ELISA binding to either (**a**) pre-fusion F or (**b**) post-fusion F. **c** Sera were also tested for virus neutralization. ELISA titers refer to serum dilution that yielded 50% of the maximal (saturating) value and neutralization titers refer to dilution that inhibited 50% fluorescence intensity. Mean ELISA titers and mean neutralization titers for each group are shown by short horizontal red lines. Long horizontal red lines in each panel indicate detection limits

Lastly, the immunogenic potential of the pre-fusion and post-fusion forms of hMPV F was evaluated. Groups of five BALB/c females were inoculated twice (4 weeks apart) with 10 µg per dose of each protein adjuvanted with CpG oligodeoxynucleotides. One week after the last dose, mice were bled and their sera tested for binding to either pre-fusion or post-fusion F and for neutralization (Fig. 7). Antibodies present in all sera bound similarly to pre-fusion and post-fusion F and equally neutralized virus infectivity, further supporting the notion that pre-fusion and post-fusion hMPV F share most neutralizing epitopes. This result is in stark contrast to that previously reported for hRSV, where the pre-fusion conformation of F elicited substantially higher titers of neutralizing antibodies than did the post-fusion conformation^26, 27, 50^. We speculate that the larger glycan shield at the apex of pre-fusion hMPV F may be responsible for this discrepancy.

## Discussion

The engineering, structure, and immunogenic features of the cleaved trimeric pre-fusion hMPV F ectodomain are presented. A single proline substitution in combination with modifications to the fusion-activating trypsin cleavage site and the addition of a foldon trimerization domain at the C-terminus of the hMPV F ectodomain generated a soluble construct with antigenic characteristics of the pre-fusion conformation. X-ray diffraction studies resulted in a 2.6 Å resolution structure, which revealed several features of pre-fusion F with important implications for its biological activity.

Notably, the fusion peptide of hMPV F resides inside the protein trimer, and the N-terminus appears to be locked into an inter-protomeric pocket by Phe456 in HRB (Fig. 3). This architecture suggests that HRB needs to be destabilized prior to release of the fusion peptide and refolding into the post-fusion conformation. Such a model would be consistent with studies on the PIV5 paramyxovirus F protein, which demonstrated that HRA peptides inhibit a temperature-arrested intermediate, suggesting that dissociation of the HRB is one of the earliest refolding events^51^. However, the pre-fusion structures of paramyxovirus F proteins from PIV5, Hendra, and Nipah all have their fusion peptides located on the periphery of the molecule, rather than buried within the central cavity, as for the pneumovirus F proteins from hMPV and hRSV^28^. Whether the location of the fusion peptide leads to differences in the mechanisms of triggering and refolding of F proteins from these two virus families remains to be determined. For hRSV F, the internal position of the fusion peptide results in a druggable pocket for therapeutic small-molecule fusion inhibitors whose mechanism of action relies on increasing the stability of pre-fusion F by tethering the fusion peptide to HRB^52, 53^. The structural similarity with pre-fusion hMPV F suggests that similar inhibitors could be isolated against hMPV, providing new opportunities for interventions against this pathogen.

Another salient feature of the pre-fusion hMPV F structure is a pH sensor that is at least partly responsible for the observed pH-sensitive fusion of certain strains of hMPV^21–25^. The sensor was observed in a parallel β-strand interaction (β1–β22) that must dissociate to allow formation of the post-fusion structure. Protonation of His435 is predicted to cause histidine–cation electrostatic repulsion, which could lead to local destabilization and initial melting of β22 from β1, but may also lead to global destabilization of the fusion glycoprotein. Although all hMPV F protein sequences contain the residues involved in the pH sensor (Lys20, Glu433, and His435), pH sensitivity is a rare strain-dependent phenomenon that is conferred by a tetrad of residues (Gly294, Lys296, Trp396, and Asn404)^22, 24^. Recent experiments have demonstrated that hMPV virions are internalized through clathrin-mediated endocytosis, and although low pH exposure modestly increases infection of strains harboring pH-sensitive F proteins, fusion is not triggered by low pH^54^. Therefore, an additional mechanism is thought to be required for hMPV F proteins to trigger at the proper time and place, such as binding to a host-cell receptor.

The hMPV F RGD motif, which has been implicated in integrin binding during the entry process^15, 16, 55^, is inaccessible in the pre-fusion conformation due to steric occlusion by neighboring protomers (Supplementary Fig. 5). Thus, trimer dissociation may be required for integrins to bind to hMPV F. Although trimer dissociation is not thought to be a general property of the fusion mechanism for class I fusion proteins^56^, it is reminiscent of intermediate steps described for class III viral fusion proteins, including VSV-G^57^. Indeed, the pH-dependent trimer-to-extended-monomer equilibrium kinetics recently observed for VSV-G^57^ offers a conceivable F-dependent mechanism for RGD-binding integrins to traffic hMPV virions into endosomes, as has been recently demonstrated^54^.

Perhaps the most unexpected differences between hMPV F and hRSV F found in this study were the specificities of human antibodies for pre-fusion and post-fusion F proteins. Whereas most hRSV-neutralizing antibodies in human Ig preparations recognized pre-fusion-specific epitopes and could not be depleted with post-fusion F, the neutralizing activity of human Igs directed against hMPV could be totally removed with pre-fusion as well as with post-fusion F (Fig. 6). The presence of the glycan shield at the apex of pre-fusion hMPV F suggests that this region, which is an immunodominant site of the neutralizing antibody response against hRSV, may be hidden in hMPV. We note, however, that despite not being evident in a polyclonal response, some apex-directed mAbs may be elicited by pre-fusion hMPV F, as suggested by at least two neutralizing mAbs with resistance mutations that map to the α4-α5 loop region^58, 59^. These intriguing immunological differences between hMPV and hRSV, and the possibility that they are due to glycan differences in their respective F proteins, will most likely have important implications for vaccine development and therefore merit further investigations.

## Methods

### Ethics statement

Animal studies were performed under the regulations of the Spanish and European legislation concerning vivisection and the use of genetically modified organisms. Protocols were approved by the “Comité de Ética de la Investigación y del Bienestar Animal” of “Instituto de Salud Carlos III (ISCIII)” (CBA PA 19_2012).

### Antibodies

All monoclonal antibodies were produced and purified in-house, except the anti-His-tag antibody. The origin of the commercial antibodies used as secondary reagents in the ELISAs is mentioned in the corresponding sections of the “Methods.” Antibodies MF14, MF16, and MF1 were obtained from hybridoma supernatants, grown in ClonaCell HT medium (Stem Cell Technologies, cat # 03805). Antibodies MPE8, ADI-15614, and ADI-18992 were obtained from supernatants of Expi293 cells (Invitrogen) transiently co-transfected with plasmids encoding antibody heavy and light chains, and grown in Expi293 expression medium (cat # A14351-01). Antibodies were purified from supernatants using ammonium sulfate precipitation followed by affinity purification over a protein A-Sepharose CL-4B column (GE Healthcare, cat # 17-0780-01) as recommended by the manufacturer. The VRC-8400 plasmid backbone encoding the heavy and light chains for MPE8, ADI-15614, and ADI-18992 was obtained from the Vaccine Research Center (VRC) at the National Institutes of Health (NIH).

### Cells

The African green monkey kidney cell line CV-1 (CCL-70TM) was obtained from ATCC and grown in Dulbecco’s modified Eagle’s medium (DMEM) supplemented with 10% fetal calf serum, 2% glutamine, and penicillin (100 units ml^-1^) plus streptomycin (100 µg ml^−1^). The monkey kidney cell line Vero-118 was a kind gift of R. Fouchier (Erasmus Medical Center, Amsterdam, Holland) and was grown in Iscove’s modified Dulbecco’s medium (IMDM) supplemented with 10% fetal calf serum, 2% glutamine, and penicillin (100 units ml^-1^) plus streptomycin (100 µg ml^−1^). All media and supplements were obtained from Lonza (Spain). Cells periodically tested mycoplasma-free using the Mycoplasma PCR ELISA kit as recommended by the manufacturer (Sigma-Aldrich). None of the cells used in this study are listed in the databases of commonly misidentified cell lines maintained by ICLAC.

### Stabilization and production of pre-fusion hMPV F variants

The pRB21 plasmid^60^ encoding the hMPV F ectodomain (amino acids 1–489) from strain NL/1/00 (A1 sublineage), stabilized in its post-fusion conformation (A1-post) and carrying the G294E mutation, has been described^34^ (Supplementary Table 1, A1-post). The related pRB21 plasmids encoding the 02-MA1 or 114-BV proteins that differed in their cleavage-site sequences (Supplementary Table 1), together with the plasmid encoding A1-post, were used for the generation of all variants listed in Supplementary Table 1. pBR21was a gift of Dr. R. Blasco (Instituto Nacional de Investigación y Tecnología Agraria y Alimentaria (I.N.I.A.), Madrid, Spain). Mutants were obtained with the Phusion site-directed mutagenesis kit (Thermo Fisher Scientific) and custom-designed primers, as recommended by the manufacturer. Information about primer sequences can be obtained from the authors upon request. All variants were confirmed by sequencing. The foldon trimerization domain^61^ was added at the C-terminus of the F protein ectodomain, flanked upstream by a TEV protease site and downstream by a Xa protease site, followed by a 6xHis-tag.

Plasmids encoding the variants listed in Supplementary Table 1 were tested for transient expression as follows: monolayers of CV-1 cells growing in DMEM medium supplemented with 10% fetal calf serum were infected at an m.o.i. of 10 p.f.u. per cell for 2 h with the furin-expressing vaccinia virus VV:bfur^62^ (a kind gift of M. Ramos, Madrid, Spain) that provided the vaccinia RNA polymerase, followed by transfection with 5 µg mL^−1^ of each plasmid mixed with 15 µg mL^−1^ of lipofectamine 2000 (Invitrogen). Forty-eight hours later, supernatants were harvested and the presence of hMPV F was detected by western blot with an anti-His-tag mAb (Bio-Rad, cat # MCA1396) or by reactivity in ELISA with the mAbs indicated in Supplementary Table 1. The anti-His-tag mAb was diluted 1:5000 or 1:3000 for western blot or ELISA, respectively.

Vaccinia virus recombinants encoding selected F protein variants (Supplementary Table 2) were obtained by the method of Blasco and Moss^60^. Proteins were produced by infecting CV-1 cells with the matching recombinant vaccinia virus (0.05–0.10 p.f.u. per cell) and co-infected with five to tenfold less of the furin-expressing vaccinia virus VV:bfur to promote cleavage, except in the case of vaccinia virus encoding 130-BV protein that lacks the furin motif. Forty-eight hours after infection, supernatants were harvested and clarified by low-speed centrifugation. After concentration and buffer exchange, proteins were purified using Ni^2+^ columns followed by gel filtration on a HiLoad 16/600 Superdex 200 pg column, as described^34^. Protein purity and integrity were checked by SDS-PAGE under reducing conditions and Coomassie blue staining.

### Electron microscopy

Purified proteins were applied to glow-discharged carbon-coated grids and negatively stained with 1% aqueous uranyl formate. Images were recorded with a Gatan ERLANGHEN 1000 W CCD camera in a JEOL JEM-1011 electron microscope operated at 100 kV at a detector magnification of ×20,400 (Fig. 1c) or a FEI Eagle CCD camera in a Tecnai G2 electron microscope operated at 200 kV at a detector magnification of ×69,444.

### Surface plasmon resonance

Antibodies were purified using Protein A-Sepharose as recommended by the manufacturer (GE Healthcare) and their antigen-binding fragments were generated by papain digestion (mass ratio, 100:1) for 4 h at 37 °C. The Fabs were bound to columns of either anti-mouse kappa or anti-human CH1 (Thermo Scientific), eluted with 0.1 M glycine, pH 3.0 and neutralized by saturation with Tris.

All assays were run in a Biacore X100 instrument. An anti-6xHis mAb was covalently bound to both the sample and reference cells of a CM5 chip at 10,000 response units (RU). Approximately 140 RU of the F proteins indicated in the figure legends were captured by the anti-6xHis mAb and then the Fabs were injected sequentially with eight to ten different protein concentrations (0.5–500 nM). Binding data were fit to a 1:1 Langmuir binding model for calculation of kinetic parameters.

### Crystallization and X-ray data collection

hMPV F construct 115-BV was expressed and purified as indicated above, and buffer exchanged again over a Superdex 200 Increase gel-filtration column (GE Healthcare Biosciences) with running buffer of 2 mM Tris pH 8.0, 200 mM NaCl. The protein was concentrated to 5.5 mg mL^−1^, and initial crystals were obtained via sitting-drop vapor diffusion from a reservoir solution of 1 M Li_2_SO_4_, 0.5 M (NH_4_)_2_SO_4_, and 0.1 M tri-sodium-citrate at pH 5.6. These crystals were further optimized by hanging-drop vapor diffusion by mixing 1 µL of protein with 1 µL of reservoir solution containing 0.82 M Li_2_SO_4_, 0.41 M (NH_4_)_2_SO_4_, and 0.1 M tri-sodium-citrate at pH 5.5. A single cube-shaped crystal was then transferred to a solution of 3.15 M ammonium sulfate and 0.1 M citrate at pH 5.5 and flash-frozen in liquid nitrogen. X-ray diffraction data were collected to 2.6 Å resolution at the AMX 17-ID-1 beamline (NSLS II, Brookhaven National Laboratory).

### Structure determination

X-ray diffraction images were indexed and integrated in iMOSFLM^63^. Reflections were then scaled and merged in AIMLESS^64^. A molecular replacement solution with a single protomer of pre-fusion hMPV F in the asymmetric unit was acquired in PHASER^65^ using a search model comprising domain II of the post-fusion hMPV F structure (PDB ID: 5L1X)^34^ complemented with the high-resolution structure of pre-fusion hRSV F (PDB ID: 5C69)^26^ substituted with the 115-BV sequence using the SCULPTOR^66^ model-editing software in PHENIX^67^. The structure was built manually in COOT^68^ and was refined in PHENIX. Data collection and refinement statistics are presented in Supplementary Table 3.

### Mouse immunization

Groups of 6-week-old female BALB/c mice (five mice per group) were inoculated twice (4 weeks apart) i.m. in the quadriceps muscles (50 µl per site) with 10 µg of the proteins in PBS, mixed with an equal volume of CpG (Magic Mouse adjuvant, Creative Diagnostics, cat # CDN-A001). One week after the last dose, mice were euthanized, their blood collected, and serum was obtained after coagulation. Sera were tested for antibody binding in ELISA and for inhibition of virus infectivity in a microneutralization test (see below). The number of mice per group is similar to that used in many other studies, including ours^69^. The mice were randomized in groups without any specific criteria. There were no inclusion or exclusion criteria, except that they were specific-pathogen-free, (seronegative for hRSV and hMPV), female BALB/c mice, each weighing ~20 g. Mice were obtained from Charles River UK Ltd. The investigators were not blinded to the groups of mice.

### Enzyme-linked immunosorbent assay

We used three different ELISA formats. (1) Capture of antigen with purified mAbs was used for testing reactivity of culture supernatants or purified proteins with mAbs. Purified mAb (400 ng) was used to coat 96-well microtiter plates. Non-specific binding was blocked with 1% BSA in PBS. Then serial dilutions of culture supernatants or purified proteins were added to the wells for 1 h at room temperature, followed sequentially by an excess of anti-His-tag mAb (Bio-Rad), streptavidin-peroxidase, and substrate (OPD, Sigma). Extensive washing with water followed each step. Optical density was read at 492 nm. (2) Capture with anti-foldon mAb was used to evaluate reactivity of human Ig preparations of FG or RG with purified F proteins. 96-well plates were coated with 400 ng of a purified proprietary murine mAb specific for the foldon trimerization domain (MF4), followed sequentially by 40 ng of purified proteins, 50 µl of commercial human Ig dilutions of peroxidase-conjugated anti-human secondary antibody (GE Healthcare), and substrate (OPD). Optical density was read at 492 nm. Where specified, human Igs were depleted of antibodies binding to F proteins indicated in the figure legends, before being used in the ELISA as follows: 45 µg of purified F protein were bound to 300 µl of Ni^2+^ resin (Sigma, P6611) by overnight incubation at 4 °C with rotation in 50 mM phosphate, pH 8.0, 300 mM NaCl, and 10 mM imidazole. After centrifugation and washing with the same buffer, 900 µg of purified Ig was added to the protein-coated resin and incubation continued for 1 h at room temperature with shaking. After centrifugation, the supernatant was saved and used in the ELISA. (3) Capture with human mAb was used to evaluate reactivity of murine sera with purified F proteins. Human mAb ADI-18902^31^ (400 ng) that binds to antigenic site IV of hRSV F but cross-reacts with hMPV F was used to coat 96-well plates. Then 40 ng of purified F proteins was added, followed sequentially by mouse sera dilutions, peroxidase-conjugated anti-mouse secondary antibody (GE Healthcare), and substrate (OPD). Optical density was read at 492 nm.

### Microneutralization assay

Mouse sera and human Ig were tested in a microneutralization assay, as follows: a predetermined amount of GFP-expressing recombinant viruses derived from either the NL/1/00 strain of hMPV (a kind gift of B. van den Hoogen and R. Fouchier, Erasmus Medical Centre, Rotterdam, NL) or the A2 strain of hRSV (a kind gift of M. Peeples, Columbus, Ohio, USA) was mixed with serial dilutions of sera or Ig before being added to cultures of Vero-118 cells growing in flat-bottom microtiter plates. Twenty-four to 48 h later, the medium was replaced by PBS, and GFP fluorescence was measured in a Tecan microplate reader M200. Values were expressed as percent of a virus control without antibody.

### Statistical analysis

Where applicable, results are expressed as the mean ± SD of the mean or as individual values and their mean. No statistical significance values are presented.

### Data availability

Coordinates and structure factors for pre-fusion hMPV F 115-BV have been deposited in the Protein Data Bank under accession code 5WB0. All other data supporting the findings of this study are available within the article and its Supplementary Information files, or are available from the authors upon request.

## Electronic supplementary material

Supplementary Information
