# Supplementary material for: Structure and immunogenicity of pre-fusion-stabilized human metapneumovirus F glycoprotein
Source: Nat Commun. 2017 Nov 16;8:1528. doi: 10.1038/s41467-017-01708-9 (PMC5688127; doi:10.1038/s41467-017-01708-9)
Supplement: Supplementary file 1 — Supplementary Information [file 41467_2017_1708_MOESM1_ESM.pdf]

Supplementary Figures

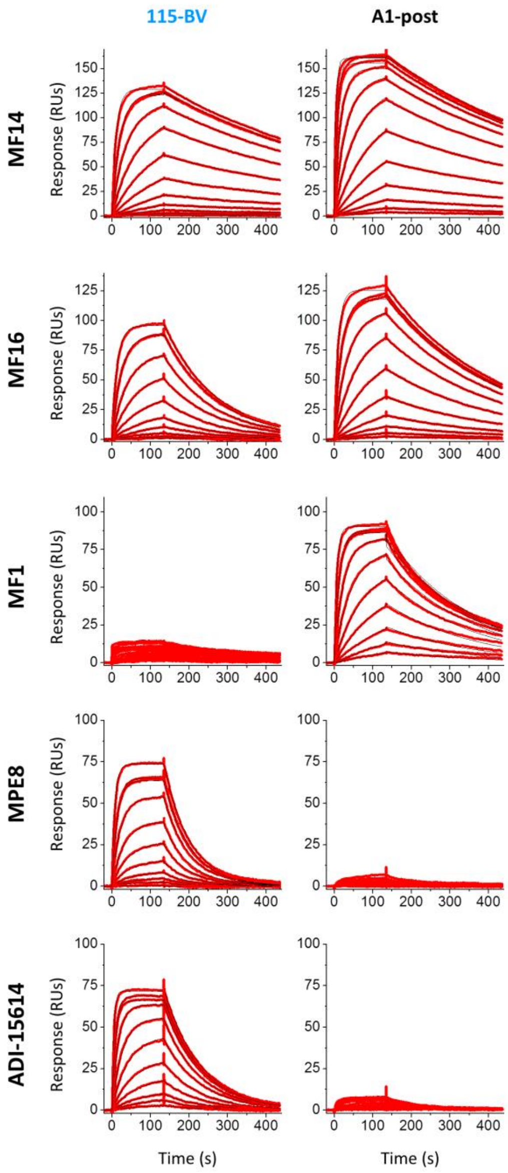

Supplementary Figure 1 | Sensorgrams for the binding of Fabs to immobilized 115-BV and A1-post proteins. Binding was measured with a Biacore X100 instrument as described in the **Online Methods**. Binding constants are shown in **Table 1**. Black traces are raw data and red traces are global fits of the data.

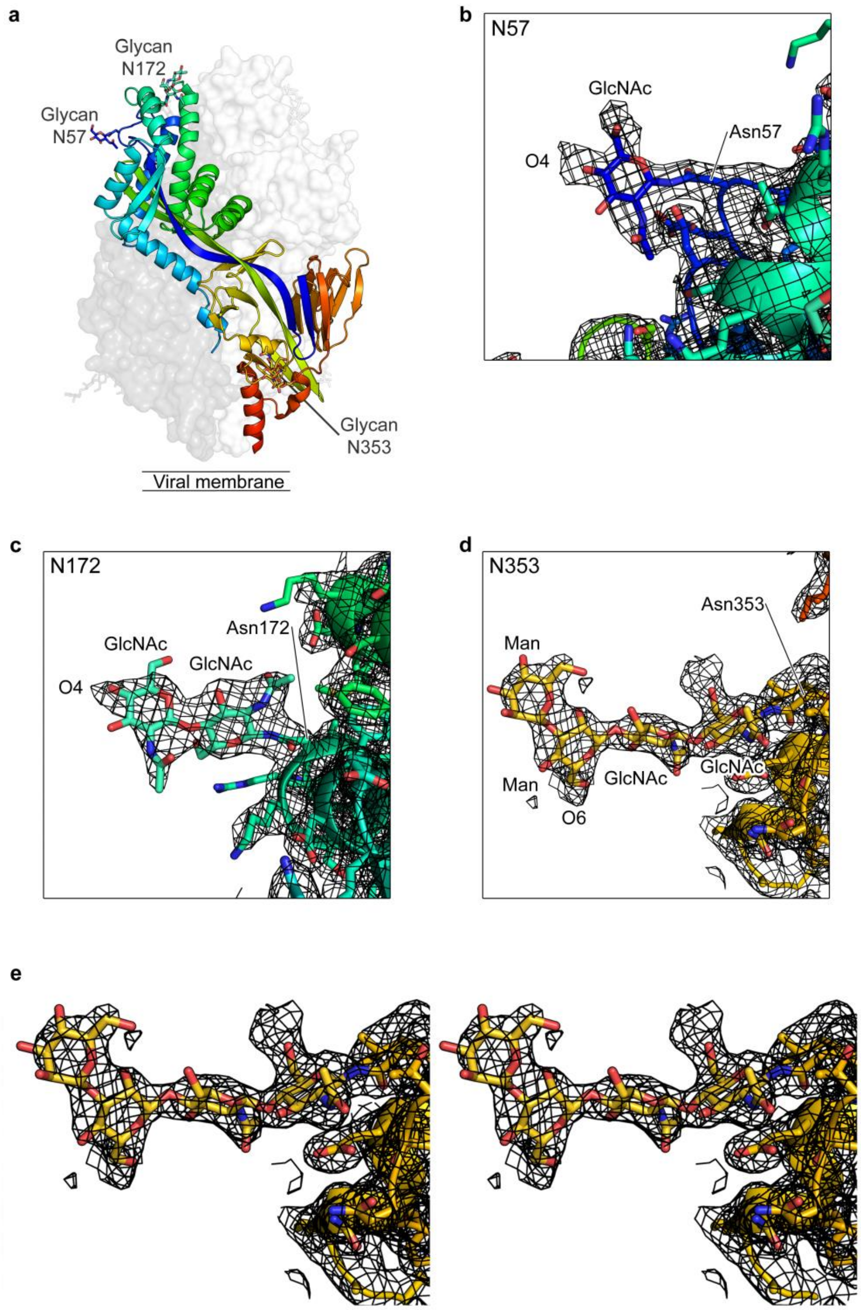

Supplementary Figure 2 | Electron density of glycans in the prefusion hMPV F crystal structure. (**a**) Side view of prefusion hMPV F as depicted in Figure 2 with gray and white protomers shown as transparent molecular surfaces. (**b–d**) Magnified regions of (**b**) N57 (**c**) N172 and (**d**) N353, colored as in (a) with glycans and proximal residues shown as sticks and with the 2*F*o-*F*c electron density map contoured at 1σ shown as a black mesh. GlcNAc = *N*-acetylglucosamine; Man = mannose. (**e**) Stereo image of N353, depicted as in (d).

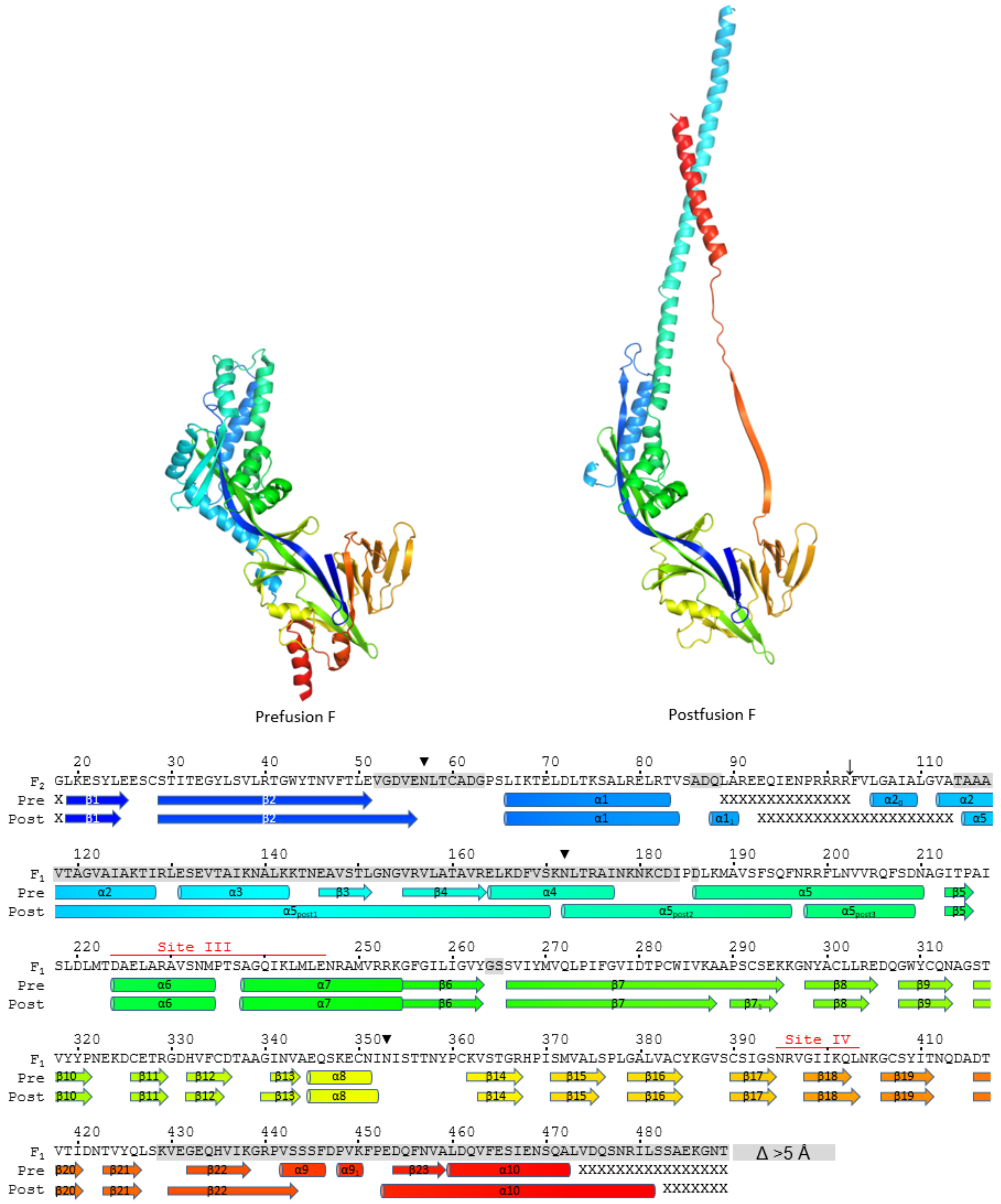

Supplementary Figure 3 | Structural rearrangement of hMPV F. hMPV F sequence and secondary structure. Sites of *N*-linked glycosylation are highlighted by black triangles, antigenic sites shared with hRSV are labeled in red, and an arrow indicates the protease cleavage site. Secondary structures are shown below the sequence, with cylinders representing α-helices and arrows representing β-strands. Disordered or missing residues are indicated by an "X"; residues that move over 5 Å between prefusion and postfusion conformations are shown with a gray shadow. The prefusion sequence of 115-BV shown above has a modified cleavage site (RQSR → RRRR) and an alanine to proline substitution (A185P).

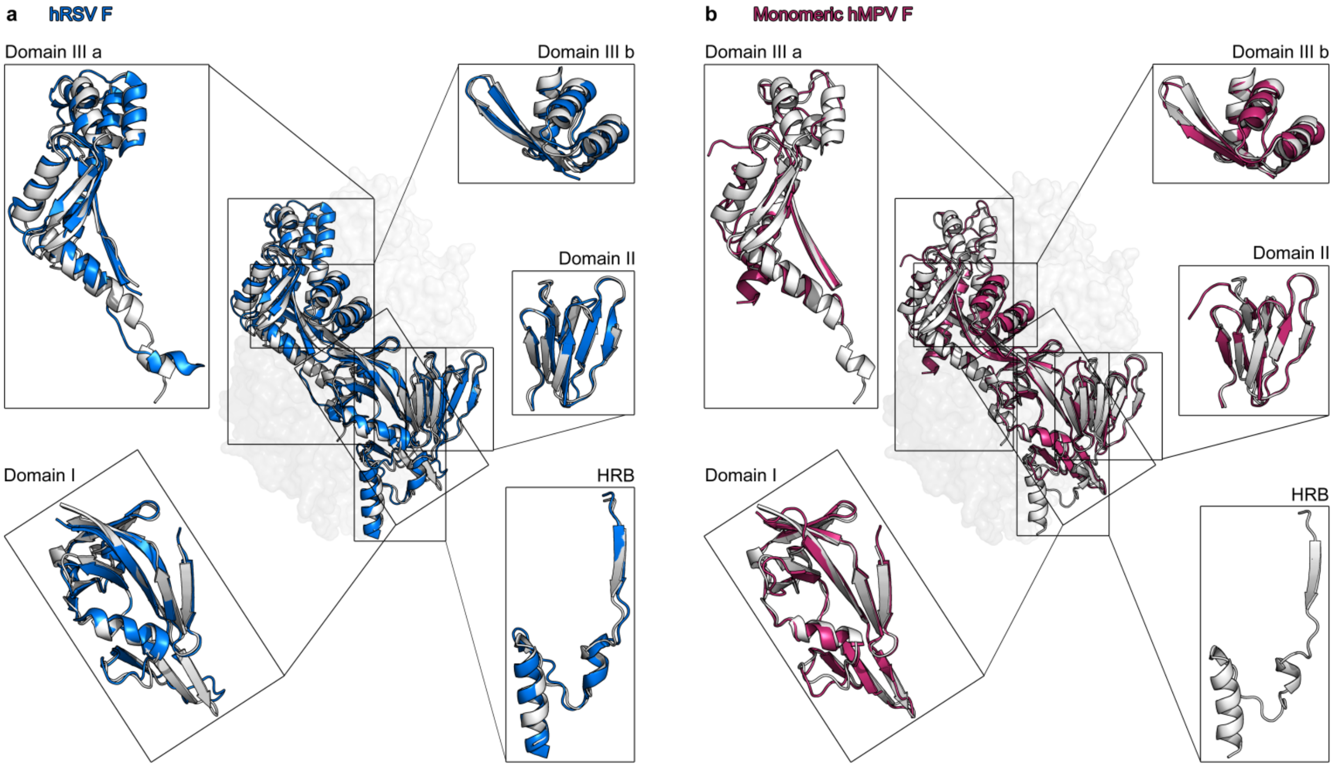

Supplementary Figure 4 | Comparison of pneumovirus prefusion F structures. Superposition of one protomer of the prefusion hMPV F structure (white) with (**a**) hRSV F (PDB ID: 5C69) and (**b**) monomeric hMPV F (PDB ID: 4DAG). Domain of the corresponding structures are superposed and magnified. Domain III has been split into subdomains that are superposed based on the full domain III alignment.

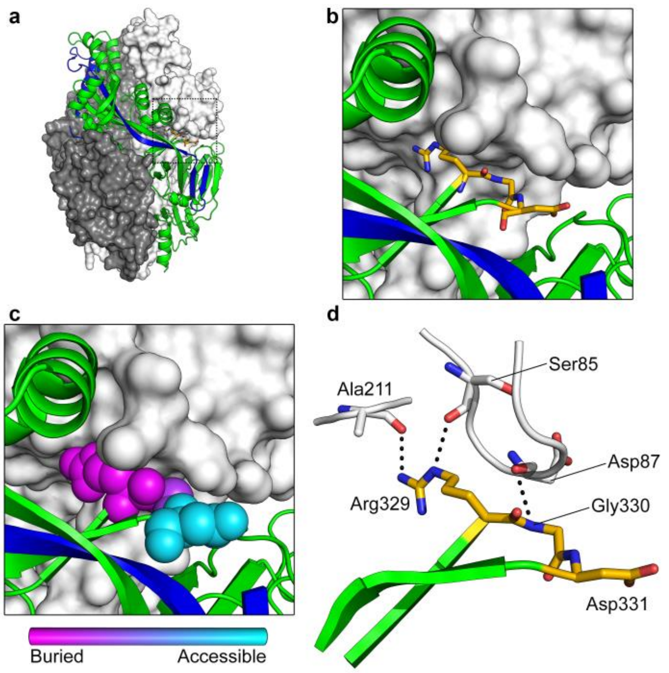

Supplementary Figure 5 | The RGD motif is mostly buried in prefusion hMPV F. (**a**) Side view of prefusion hMPV F with (**b**) magnified region depicting the RGD motif in gold. (**c**) Fraction of accessible surface area buried by neighboring protomer for the RGD motif colored on a per-residue basis: magenta (0%, "buried") to cyan (100%, "accessible"). In the prefusion hMPV F structure, Arg329, Gly330 and Asp331 have 125, 61, and 63 Å^2 of accessible surface area, of which 117, 45 and 6 Å^2 are buried by the neighboring protomer, respectively. (**d**) Magnification of RGD motif showing interprotomeric hydrogen bonds.

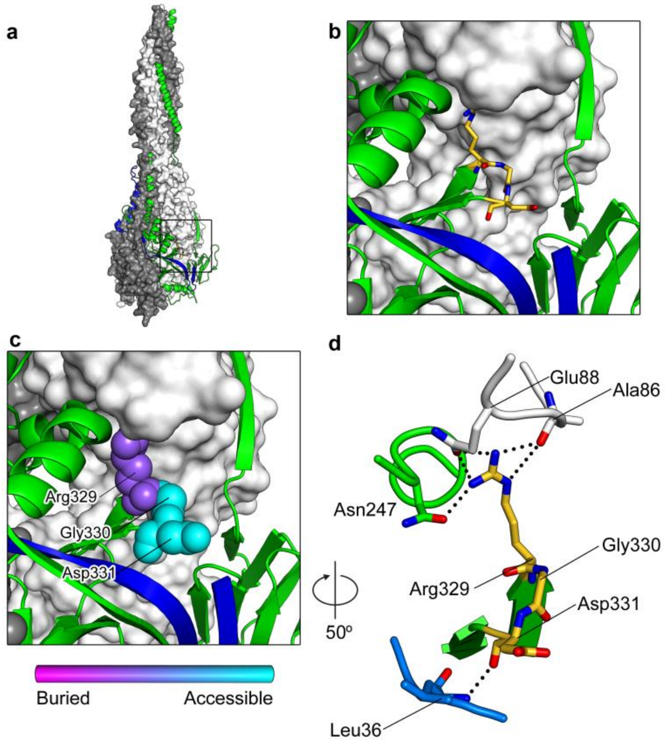

Supplementary Figure 6 | The RGD motif is partially buried in postfusion hMPV F. (**a**) Side view of postfusion hMPV F with (**b**) magnified region depicting the RGD motif in gold. (**c**) Fraction of accessible surface area buried by neighboring protomer for the RGD motif colored on a per-residue basis: magenta (0%, "buried") to cyan (100%, "accessible"). In the postfusion hMPV F structure, Arg329, Gly330 and Asp331 have 114, 57, and 71 Å^2 of accessible surface area, of which 70, 0 and 0 Å^2 are buried by the neighboring protomer, respectively. (**d**) Magnification of RGD motif showing select hydrogen bonds.

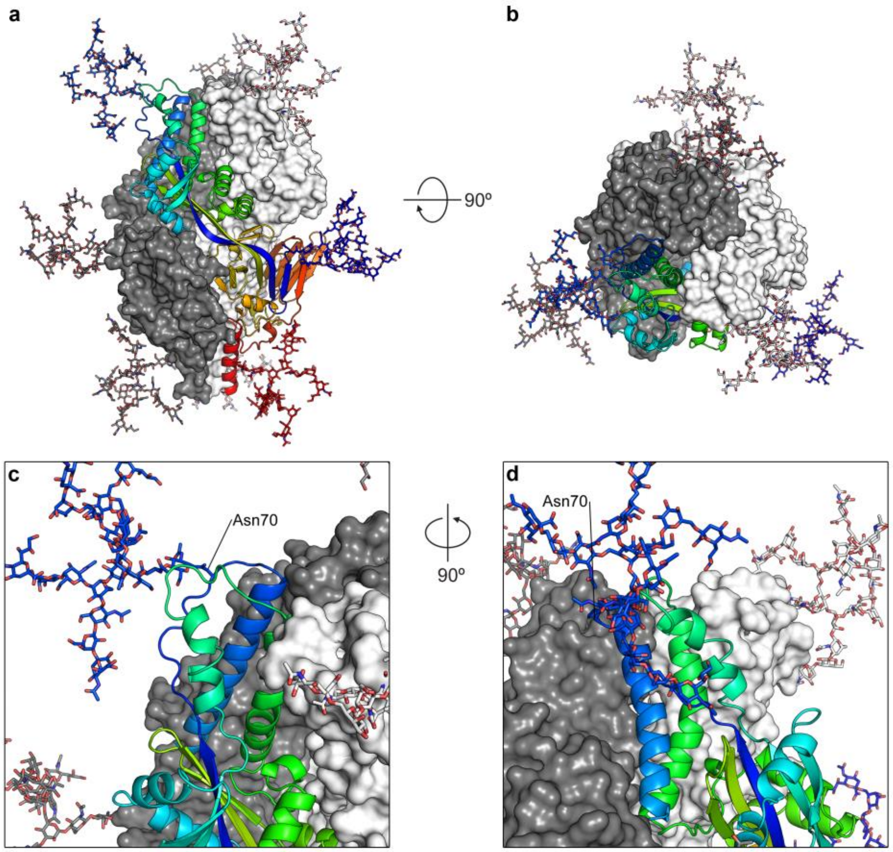

Supplementary Figure 7 | Conserved *N*-linked glycosylation in prefusion hRSV F. (**a**) Side view and (**b**) top view of prefusion hRSV F, with complex glycans (modelled *in silico* via GlyProt [1]), shown as sticks. (**c**) Magnified view of the apex, and (**d**) 90° rotation. Oxygen atoms are colored red and nitrogen atoms are colored blue.

Supplementary Table 1 | List of hMPV F glycoprotein variants

Name	Mechanism of stabilization	Cleavage site	Mutation	Efficiency of cleavage (WB)	Supernatant volume (50% Max, µl) MF14 (Pre & Post)	MF1 (Post)	MPE8 (Pre)
A1-post		ENPRQS**KKRKRR**	Δ103-111	**> 95%**	**6.80**	**5.14**	**18.32**
02-MA1		ENP**RQSR**		< 5%	6.27	> 50	7.77
114-BV		ENP**RRRR**		> 95%	8.65	28.76	9.26
115-BV	α4-α5 hinge	ENP**RRRR**	A185P	**> 95%**	**1.99**	**14.83**	**2.54**
116-BV		ENP**RRRR**	I184P	> 95%	10.60	20.81	28.33
117-BV		ENP**RRRR**	D186P	> 95%	13.03	> 50	11.97
130-BV		ENP**RQSR**	A185P	**< 5%**	**0.69**	**> 50**	**0.89**
134-BV		ENPRQS**KKRKRR**	A185P	> 95%	6.16	30.12	11.25
135-BV		ENP**RTKR**	A185P	≈ 50%	1.07	36.99	1.60
136-BV		**TD**P**RTKR**	A185P	≈ 75%	2.23	20.55	2.94
137-BV		ENPRQS**KKRKRR**	Δ103-111 A185P	> 95%	1.10	2.82	2.41
118-BV	Cavity filling	ENP**RRRR**	F456W	**> 95%**	**5.43**	**> 50**	**5.51**
119-BV		ENP**RRRR**	A161F	ND	> 50	> 50	> 50
120-BV		ENP**RRRR**	A161L	> 95%	33.33	> 50	25.64
121-BV	Disulfide formation	ENP**RRRR**	T127C/I258C	ND	> 50	> 50	> 50
122-BV		ENP**RRRR**	T127C/I260C	ND	> 50	> 50	> 50
123-BV		ENP**RRRR**	A120C/F256C	ND	> 50	> 50	> 50

The names of the different hMPV F proteins used in this study are indicated. Plasmids encoding the first three proteins (which fold spontaneously into the postfusion conformation) were the basis for generating the remaining mutants that differ in the indicated cleavage site sequence and in the mutations intended to stabilize them in the prefusion conformation. Plasmids were tested for transient expression of the encoded proteins in CV-1 cells, as described in the **Methods**. The presence of the different proteins in culture supernatants was assessed by western blot (which also informed about the degree of cleavage) and by ELISA with mAb MF14 that recognizes an epitope shared by the prefusion and postfusion conformations [2]. ELISA with MF1 [3] and MPE8 [4] provided information about reactivity with postfusion and prefusion specific mAbs, respectively. Numbers indicate microliters of culture supernatant required to reach 50% of maximum value in ELISA. Those in boldface indicate proteins that have been produced and purified in this study. ND, not detected.

Supplementary Table 2 | Characteristics of purified hMPV F glycoproteins

Name	Mechanism of stabilization	Cleavage site	Mutation	Yield mg/L	Oligomerization state	Trimer Form (EM)	Efficiency of cleavage (SDS-PAGE)	ELISA EC50 (nM) MF14	MF1	MPE8	ADI-18992	ADI-15614
A1-post		ENPRQS**KKRKRR**	Δ103-111	0.4	Trimers & minor aggregates	Post	**> 95%**	**2.01**	**2.37**	**18.5**	**6.80**	**26.1**
115-BV	α4-α5 hinge	ENP**RRRR**	A185P	1.0	Trimers & minor aggregates	Pre	**> 95%**	**1.21**	**64.0**	**1.75**	**4.71**	**1.82**
130-BV	α4-α5 hinge	ENP**RQSR**	A185P	1.7	Trimers	Pre	**< 5%**	**1.25**	**> 100**	**1.84**	**3.71**	ND
118-BV	Cavity filling	ENP**RRRR**	F456W	0.3	Trimers	Pre & Post	**> 95%**	**2.56**	**11.6**	**6.13**	ND	ND

Proteins were produced and purified as described in the **Methods**. Purity and efficiency of cleavage was evaluated by SDS-PAGE with Coomassie blue staining, and oligomerization state was determined by gel filtration. The indicated mAbs were used to capture the proteins that were assessed by ELISA as described in the **Methods**. ADI-15614 and ADI-18992 are human mAbs specific for antigenic site III (unique to the prefusion conformation) and site IV (shared by prefusion and postfusion) of hRSV F, respectively, but that cross-react with hMPV F [5]. Other mAbs, as in **Supplementary Table 1**. ND, not determined.

Supplementary Table 3 | Crystallographic data collection and refinement statistics

	Prefusion hMPV F
**Data collection**	
Space group	$I2_13$
Cell dimensions	
a=*b*=*c* (Å)	177.7
α=β=γ (°)	90
Resolution (Å)	51.3–2.6 (2.72–2.60)*
R_{merge}	0.165 (1.42)
$I / \sigma I$	11.2 (1.90)
Completeness (%)	100.0 (100.0)
Redundancy	9.9 (10.3)
**Refinement**	
Resolution (Å)	51.3–2.60
Unique reflections	28,796 (1,455)
R_{work} / R_{free} (%)	17.0/20.7
No. atoms	
Protein	3,355
Carbohydrate	92
Ion	520
Water	135
B-factors (Å^2)	
Protein	57.8
Carbohydrate	116.0
Ion	143.9
Water	52.4
R.m.s. deviations	
Bond lengths (Å)	0.003
Bond angles (°)	0.56

Data were collected from one crystal.

*Values in parentheses are for highest-resolution shell.

Supplementary References

1. Bohne-Lang, A. & von der Lieth, C.W. GlyProt: in silico glycosylation of proteins. *Nucleic Acids Res* **33**, W214-9 (2005).
2. Mas, V. et al. Engineering, Structure and Immunogenicity of the Human Metapneumovirus F Protein in the Postfusion Conformation. *PLoS Pathog* **12**, e1005859 (2016).
3. Rodriguez, L. et al. Generation of monoclonal antibodies specific of the postfusion conformation of the Pneumovirinae fusion (F) protein. *J Virol Methods* **224**, 1-8 (2015).
4. Corti, D. et al. Cross-neutralization of four paramyxoviruses by a human monoclonal antibody. *Nature* **501**, 439-43 (2013).
5. Gilman, M.S. et al. Rapid profiling of RSV antibody repertoires from the memory B cells of naturally infected adult donors. *Sci Immunol* **1**(2016).
